# New species and records of *Cloeodes* Traver, 1938 (Ephemeroptera, Baetidae) from Costa Rica

**DOI:** 10.3897/zookeys.989.53018

**Published:** 2020-11-09

**Authors:** Oscar Vásquez-Bolaños, Fabián Sibaja-Araya, Meyer Guevara-Mora

**Affiliations:** 1 Laboratorio de Entomología (LEUNA), Escuela de Ciencias Biológicas, Universidad Nacional, Heredia, Costa Rica Universidad Nacional Heredia Costa Rica

**Keywords:** diversity, freshwater, mayflies, Neotropics, taxonomy

## Abstract

The nymph of *Cloeodes danta***sp. nov**. is described from male and female nymphs collected from highland streams in the Caribbean Slope of the Costa Rica Central Volcanic Mountain Range. Adults are unknown. In addition, *C. excogitatus* and *C. redactus* are recorded for the first time in the country. *Cloeodes danta***sp. nov.** can be differentiated from all described species by the predominantly brownish coloration on females and a similar coloration on males but with segments VII–IX light yellow and light brown, with no conspicuous marks or patterns; abundant scale-bases throughout most parts of the body; hindwings pads absent; the presence of three spines in the corners of the posterior margin of sternum III, and the posterior margin of tergum III with 28–30 spines on each side of the middle line (spine with a base width up to 0.5× spine length).

## Introduction

The genus *Cloeodes* (Ephemeroptera, Baetidae) was erected by Traver (1938) for a group of species described from Puerto Rico with hind wings absent. Later, the genus was reviewed by Waltz and McCafferty (1987) and established for other species distributed across the Neotropics, Southern Nearctic, Afrotropics, and Oriental region. More recently, through phylogenetic analysis the genus has been depicted as exclusive for the New World within a complex including *Bungona* spp., *Crassolus* spp. and *Potamocleoen* spp. (Salles et al. 2016). Currently, the number of *Cloeodes* species has doubled in the last ten years with 26 species described for the Neotropics (Salles et al. 2016; Kluge 2017).

Despite the increase in the number of papers related to *Cloeodes* and the report of 142 mayfly species in Central America and the Caribbean (Cornejo and Flowers 2015; Salles et al. 2016), there are only two *Cloeodes* species formally reported from the Central American region: *C. excogitatus* (Waltz & McCafferty, 1987) and *C. redactus* (Waltz & McCafferty, 1987), registered from Guatemala and Honduras, respectively (McCafferty and Lugo-Ortiz 1996; McCafferty et al. 2004). In Costa Rica, the presence of the genus has been recorded for lowland and midland streams with scarce human intervention and good water quality (McCafferty et al. 1992; Flowers and De la Rosa 2010), but no species have been formally reported until now.

During an ongoing study of the richness and taxonomy of the family Baetidae in Costa Rica, nymphs of a new *Cloeodes* species and nymphs of *C. excogitatus* and *C. redactus* were collected in low order streams. This work provides a complete description of this new species and diagnostic characters for distinguishing *C. excogitatus* and *C. redactus*, in order to improve the taxonomic knowledge of the genus for future natural history or ecological studies of mayflies, which have crucial knowledge gaps in the tropics (Balian et al. 2008; Ramírez and Gutiérrez-Fonseca 2014).

## Material and methods

**Figure 1.**
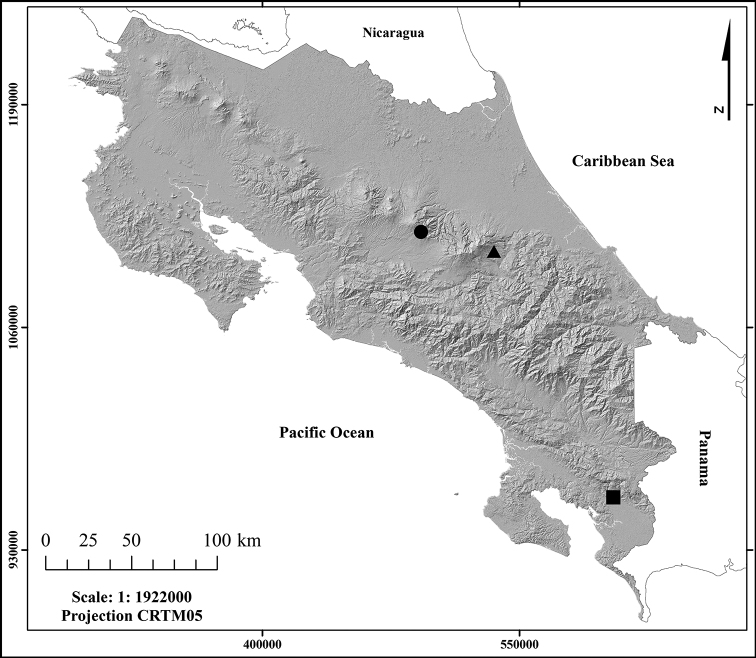
Geographic distribution in Costa Rica of *Cloeodes danta* sp. nov. (black circle), *Cloeodes excogitatus* (black triangle) and *Cloeodes redactus* (black square).

Nymphs of these species were collected using a hand net to scrape large stone substrates from different streams in Costa Rica (Fig. 1). Line drawings for the new species description were made from original pictures taken with an AmScope 1803 digital camera adapted to a Premiere (MRP–161) microscope and stereomicroscope Premiere SMZ-05. The type material examined is preserved in 80% ethanol and Euparal permanent slides, and it has been deposited in Museo de Nacional de Costa Rica, San José Province; Laboratorio de Entomología (LEUNA), Escuela de Ciencias Biológicas, Universidad Nacional, Heredia; Museo de Zoología, Universidad de Costa Rica; and Purdue University Entomological Research Collection (PERC), West Lafayette, Indiana, USA.

## Results

### Cloeodes danta

Taxon classificationAnimaliaEphemeropteraBaetidae

Vásquez-Bolaños, Sibaja-Araya & Guevara-Mora sp. nov.

CD3C133E-EC1B-589F-9DFF-0D7837F8443B

http://zoobank.org/F2F0FC62-E8EE-4E79-93F6-48F711DAC45C

**Figure 2.**
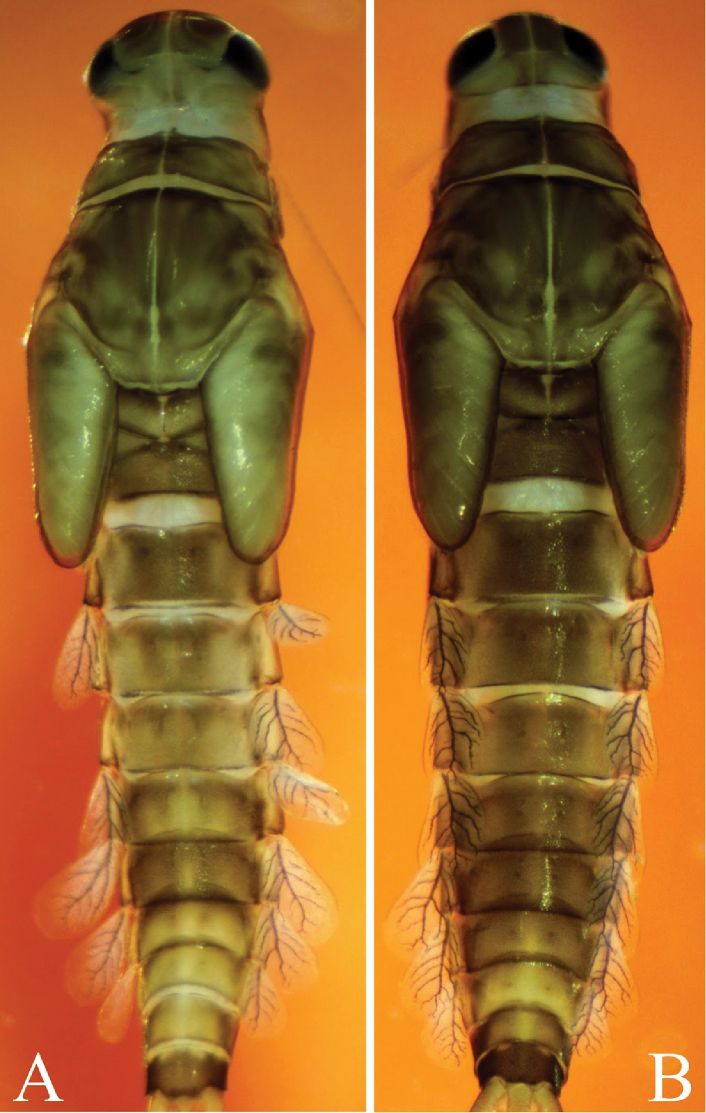
*Cloeodes danta* sp. nov. Body coloration **A** male **B** female.

**Figure 3.**
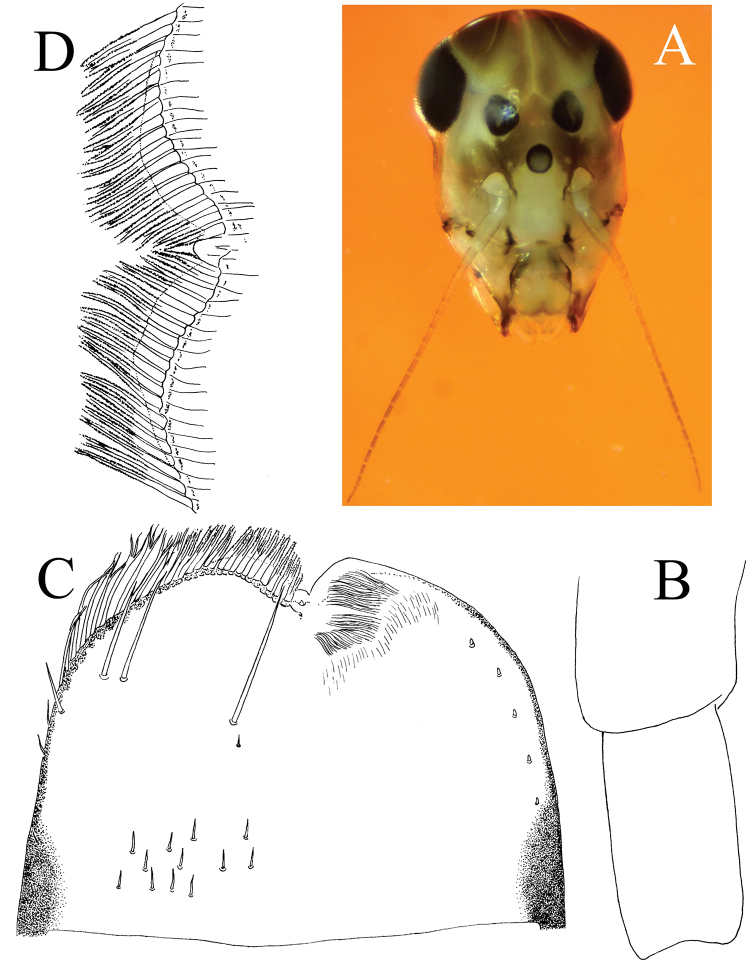
*Cloeodes danta* sp. nov. **A** head color pattern and intra-antennal extension detail **B** scape and pedicel **C** labrum (left d. v., right v. v.) **D** detail of labrum asymmetry on anterior margin.

**Figure 4.**
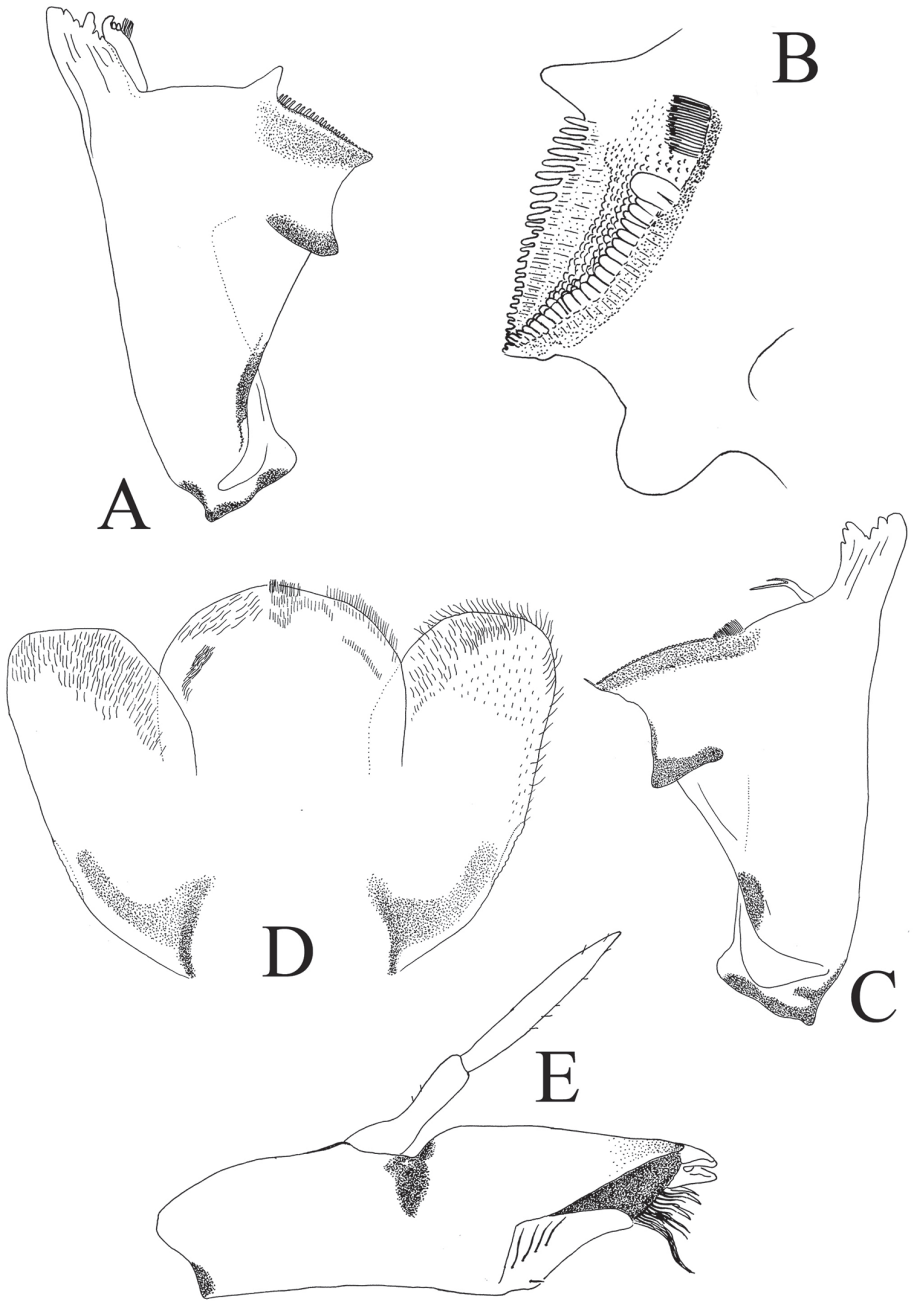
*Cloeodes danta* sp. nov. **A** left mandible **B** detail of molar region, left mandible **C** right mandible **D** hypopharynx (left d. v., right v. v.) **E** maxilla.

**Figure 5.**
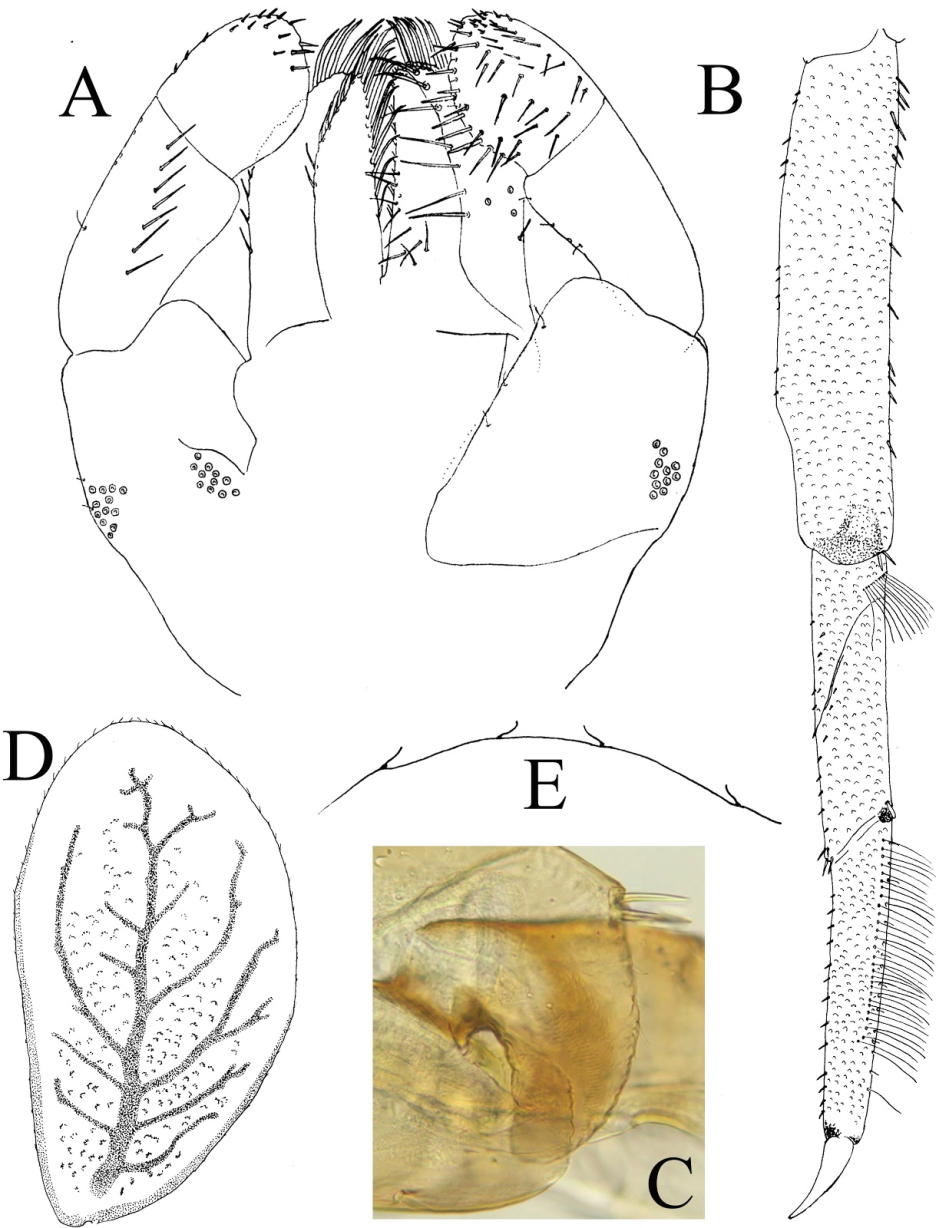
*Cloeodes danta* sp. nov. **A** labium (left d. v., right v. v.) **B** foreleg **C** forefemur apex detail **D** gill III **E** gill III apex detail.

**Figure 6.**
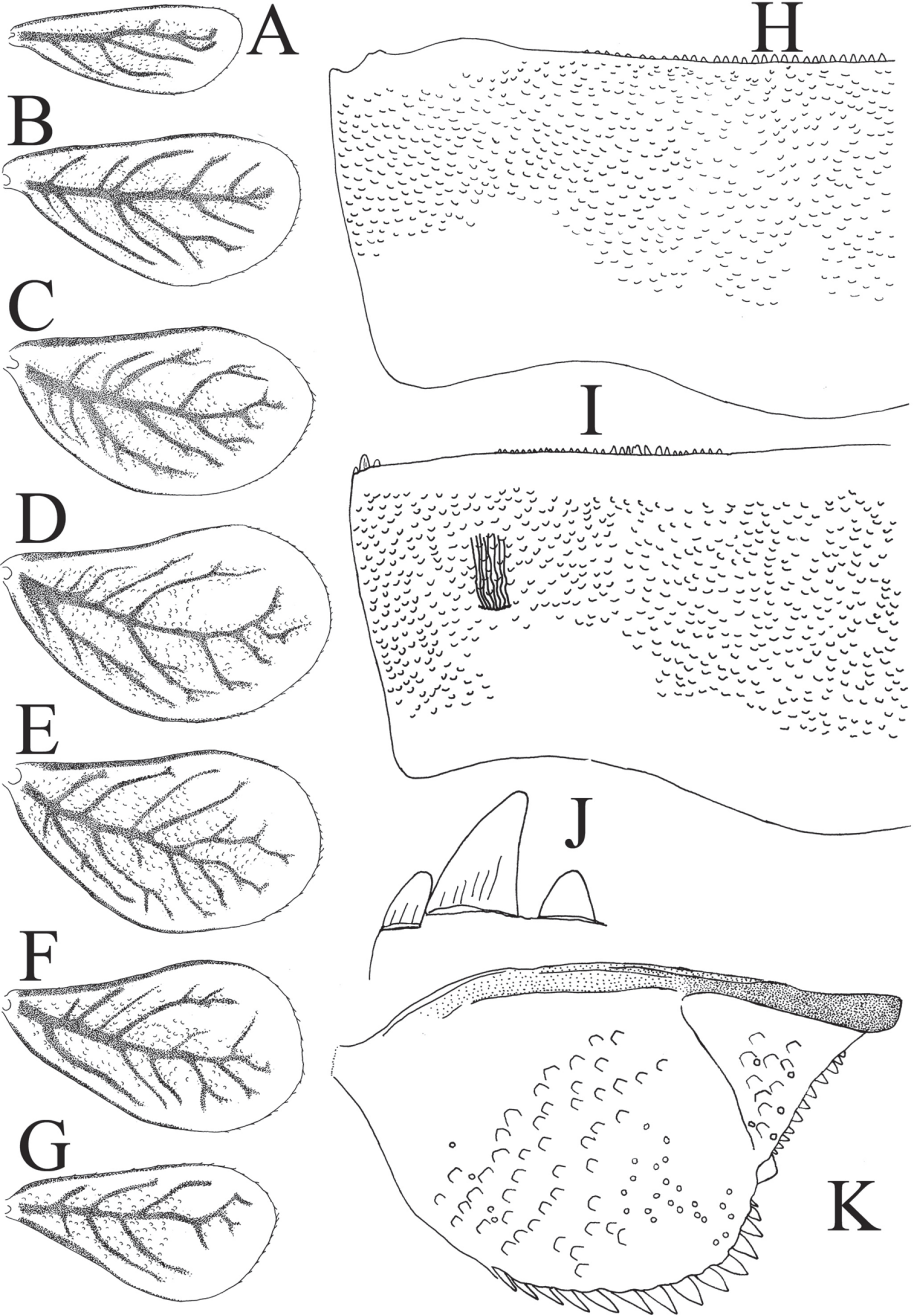
*Cloeodes danta* sp. nov. **A** gill I **B** gill II **C** gill III **D** gill IV **E** gill V **F** gill VI **G** gill VII **H** abdominal tergum III **I** abdominal sternum III **J** detail of sternum spine-like teeth **K** paraproct.

Figures 2 , 3 , 4 , 5 , 6

#### Material examined.

***Holotype***: mature ♂ nymph slide-mounted in Euparal, Costa Rica, Heredia Province, Central Volcanic Mountain Range, Cerro Chompipe zone, Refugio de Vida Silvestre Cerro Dantas, Río Las Vueltas, 10°5'38.24"N, 84°3'42.57"W, 2000 m above sea level, VI/09/2017, Sibaja-Araya F. and Acuña F. (colls), deposited in Museo de Nacional de Costa Rica, San José Province. ***Paratypes***: 5 mature nymphs same data as holotype, preserved in 95% alcohol (mouthparts, legs, gills, terga and paraprocts in micro-vial) deposited at Laboratorio de Entomología (LEUNA), Escuela de Ciencias Biológicas, Universidad Nacional, Heredia (1♂, 1♀); Museo de Zoología, Universidad de Costa Rica (1♀) and PERC, West Lafayette, Indiana, USA (1♂, 1♀).

#### Additional material.

Nine nymphs, Quebrada Grande, Refugio de Vida Silvestre Cerro Dantas, 10°5'38.24"N, 84°3'42.57"W, about 1 km from the Río Las Vueltas at 2054 m asl, where type material was collected, Heredia Province, Costa Rica V/13/2018, F. Sibaja, D. Romero, M. Guevara, D. Segura, O. Vásquez (colls), deposited at Laboratorio de Entomología (LEUNA) of the Escuela de Ciencias Biológicas, Universidad Nacional.

#### Diagnosis.

**Figure 7.**
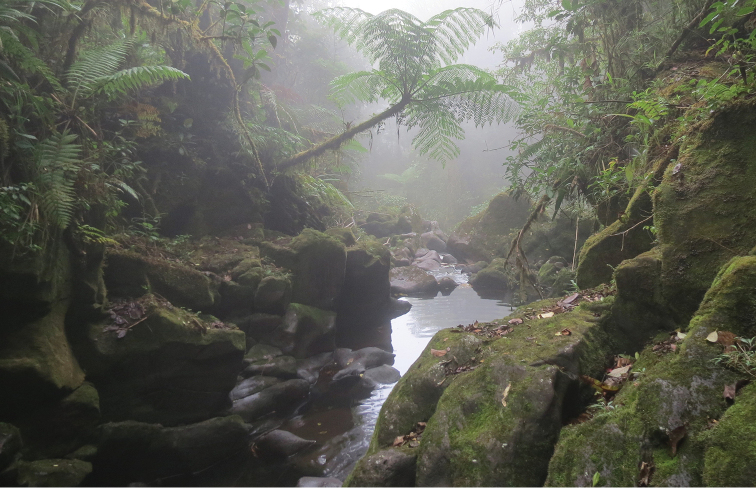
Habitat of *Cloeodes danta* sp. nov. at Río Las Vueltas.

**Mature nymph**. 1) Brownish body coloration, without conspicuous marks or patterns (Fig. 2), 2) abundant scale-bases throughout most parts of the body (Figs 5b, 6h, 6i, 6k), 3) Absence of hind wing pads, 4) Tibia with two parallel lines of fine long hairs (Fig. 5b), 5) posterior margin of tergum III with 28–30 spines on each side of the middle line (Fig. 6h), 6) sterna with 26–28 variable size spines on each side of the middle line and three thick, spine-like teeth in each corner of the posterior margin of sternum III (Fig. 6i, 6j), 7) paraproct with about 13–15 spines (Fig. 7k).

#### Description.

(based on last instar male and female nymphs; adults unknown).

**Size** (Mature nymphs): Body length: 5.3–6 mm males, 5.5–6.2 mm females; antennae 1.5–1.7 mm; cerci 2.4–2.7 mm; terminal filament 2.2–2.5 mm.

**Body coloration**: Brownish in general (Fig. 2A, 2B), the head light brownish, with clearer area from central ocelli to the border of clypeus and between antennal and labrum bases; ocelli black, with two tiny white symmetrical dots on each side and clear brownish coloration, darker toward ocellus; eyes black, turbinated portion of compound eyes brownish. Fore wing pads brownish, foreleg brown with slightly lighter areas. Abdomen with even brownish coloration on females and males with predominantly brownish color but with segments VII–IX light yellow and light brown, both with no patterns of marks or spots, and upper corners of abdominal segments darkened; terga I–VIII with visible black posterior sigilla in the middle of every segment, terga VII–IX lighter with upper corners darkened; sterna pale yellowish-brown.

**Head** (Fig. 3A): Capsule longer than wide. Antennae subequal to 1.3× length of head, with scape subequal to 1.3× times length of pedicel (Fig. 3B). Intra-antennal extension of frons prolongs to ocelli.

Labrum (Fig. 3C). Subrectangular, broader than long, anterolateral margins rounded; dorsally with anterior margin with about 20 small, double frayed setae. Lateral margin with eight apically frayed setae; arc of anterodorsal setae with four simple setae; intermediate seta tiny; and several small scattered simple setae near posterior margin. Ventrally with small curved fine setae near anterior margin, and seven small simple setae near lateral margin. Anterior margin slightly asymmetric, with the left side of the cleft not extended the same length as the right side (Fig. 3D).

Left mandible (Fig. 4A). Incisors with seven denticles, middle one reduced and others similar in size; prostheca robust, apically with three lobes and four elongated projections. Row of 5 or 6 minute spine-like setae between prostheca and molar region, only visible at high magnification (40×). Ventral surface of molar region with tuft of spines next to thumb as part of molar structure (Fig. 4B).

Right mandible (Fig. 4C). Incisors with seven denticles, middle one reduced and others similar in size; prostheca with broad base, bifid, inner projection longer, and both parts frayed; 3–4 tiny, simple setae between prostheca and molar region only visible at high magnification (40×); tuft of spines next to molar region.

Hypopharynx (Fig. 4D). Lingua rounded with no apical lobes, slightly longer and broader than superlinguae, both apically covered with short fine hairs on dorsal and ventral surface.

Maxillae (Fig. 4E). Palpi slightly shorter or as long as galea-lacinia, two segmented; segment I slender in mid part; segment II 1.4× length of segment I; both segments with several simple short setae. Crown of galea-lacinia without thick distal dentisetae, with numerous setae on the inner-dorsal and inner ventral rows, and longer and slender towards the biting edge; medial region of galea-lacinia with one short seta and 5 or 6 long, simple setae.

Labium (Fig. 5A). Glossa and paraglossa similar in length, basally broad and apically narrow; with the base of the glossa reaching more than half of the paraglossa; glossae dorsally with 7–8 setae next to the inner margin and some scattered setae, ventrally with 12 or 13 setae on the inner margin and 9 or 10 on the outer margin, increasing in length apically in both cases. Paraglossae curve inwards, apex subtriangular, dorsally with 7 or 8 setae on the inner margin and 17 or 18 setae on outer margin. Palpi with segment I broad and 0.8× length of segment II and III combined, numerous micropores and simple, tiny setae on both dorsal and ventral surfaces; segment II with row of 6 or 7 setae on the dorsal surface; segment III suboval, dorsally with several short simple setae on apex and 20–25 simple setae of different size on the ventral surface, and the inner margin subequal to the outer margin.

**Thorax**: Hindwing pads absent; foreleg with abundant scale-bases and scattered micropores (Fig. 5B). Femur length about 4× maximum with; dorsal edge with 10 or 11 short, simple setae, ventrally bare, apex rounded with no evident projections, and two concave and apically rounded setae. Tibia with two lines of fine hairs arranged parallel to each other along the tibia, subtending bristle elongated and rounded (Fig. 5B, C). Tarsi dorsal edge bare, ventrally with 10 or 11 minute spines, increasing in size distally; tarsal claw length 0.5× length of tarsi. Mid and hind legs are similar to the foreleg.

**Abdomen**: Gills (Fig. 6A–G). Present on segments I–VII, colorless, oval, margins with curved fine setae (Fig. 6D, 6E); light-brown tracheae extending from main trunk, branching to margins. Gill I smaller than segment II; gill IV length about 1.3× length of segment III; gills on segment VII not extending beyond apex of segment X (Fig. 2A, B).

Terga. Tergum I with no spines on posterior margin; tergum III with 28–30 spines on posterior margin of each side of the middle line, spine bases almost same width as height (Fig. 6J).

Sterna (Fig. 6I). Posterior margin of sternum III with group of 26–28 variable size spines on each side of middle line, spines bases width about one-third of height; group of fine hairs arranged in slightly curved line on each side of sterna and three thick spine-like teeth on each corner of posterior margin of sternum III (Fig. 6H).

Paraprocts (Fig. 6K). Lateral margin with 13–15 spines, different in size and arranged in irregular line, with some overlapping; dorsal surface with numerous micropores and scale-bases; posterolateral extension with about 11 or 12 marginal spines, and several scale-bases and micropores.

#### Adults.

Unknown.

#### Etymology.

The name of this species honors the Danta (*Tapirus bairdii*) (Mammalia: Tapiridae), a common species in the Cerro Chompipe cloud forest zone, whose three-toed back feet resemble the sternal spine-like teeth described as a diagnostic character of *C. danta* sp. nov.

#### Distribution.

Costa Rican, Caribbean slope basin, first order streams, above 2000 m asl.

#### Biology.

Habitat preferences in *C. danta* sp. nov. were observed in relation to elevation. Individuals were found at 2054 m asl in cold waters of two cloud forest streams (Fig. 7); other non-Andean species have been reported between 159–1800 m asl (Nieto and Richard 2008; Gutiérrez and Reinoso-Flórez 2010; Massariol and Salles 2011; Massariol et al. 2013; De Paul et al. 2013; Gutiérrez and Dias 2015). Also, the new species was collected in a pristine forest region, which may be similar to other *Cloeodes* species in Brazil that seem to prefer remnant forest areas (Salles et al. 2015). In addition, *C. danta* sp. nov. is common in riffles, on igneous boulders that are covered with periphyton; this is typical for this genus, part of the grazer functional feeding group (Baptista et al. 2006).

### Cloeodes excogitatus

Taxon classificationAnimaliaEphemeropteraBaetidae

(Waltz & McCafferty, 1987)

280E0FC6-BAE6-5E40-B70D-D2E46295105E

Cloeodes excogitatus Waltz & McCafferty, 1987: 200; McCafferty and Lugo-Ortiz 1996: 23; Wiersema and Baumgardner 2000: 61; McCafferty et al. 2004: 207.

#### Material examined.

***Paratype***, *Cloeodes excogitatus*, R.W. Koss and R. Baumann, 1 ♂ / Collected 12/V/1968, Arizona, Oak Creek Canyon, slide-mounted in Euparal (Abs. Alc.) by R.B. Waltz VI/1983, det. by Waltz and McCafferty 1984. (Paratype), PERC (0,012,327). *Cloeodes excogitatus*, DE Baumgardner, 1 male/ 1 female / Collected 21/V/2004, Arizona, Geenlee CO. San Francisco R. at FS Road, 212, ca 1 mi. N. Clifton. 33°04'30"N, 109°18'04"W, Elev. 3700 ft, (DB04-21), det. M. Meyer 2005. 10 mature nymphs, Río Torito, La Fuente, Santa Teresita, Turrialba, Cartago, Costa Rica, 9°59'13.04"N, 83°40'44.95"W, 1063 m asl, II/28/2017. Romero D. and Sibaja-Araya F, (colls). Material in 95% alcohol, deposited at Laboratorio de Entomología (LEUNA), Escuela de Ciencias Biológicas, Universidad Nacional, Heredia, Costa Rica.

#### Distribution.

Guatemala, Mexico, USA (Waltz and McCafferty 1987; McCafferty and Lugo-Ortiz 1996; Wiersema and Baumgardner 2000; McCafferty et al. 2004), and Costa Rica.

### Cloeodes redactus

Taxon classificationAnimaliaEphemeropteraBaetidae

(Waltz & McCafferty, 1987)

09A70DAB-D8A5-5E08-9D6C-5CFD259B5E61

Cloeodes redactus Waltz & McCafferty, 1987: 204; McCafferty and Lugo-Ortiz 1996: 23; Nieto and Emmerich 2011: 58; Massariol et al. 2013: 11; Forero-Céspedes et al. 2016: 462; Kluge 2017: 104.

#### Material examined.

Five mature nymphs, Río Claro tributary, Río Claro, Golfito, Puntarenas, Costa Rica. 8°41'13.05"N, 83°02'48.78"W, 79 m asl, XII/25/2018. Coll. Sibaja-Araya F. (coll.). Material in 95% alcohol deposited at Laboratorio de Entomología, Escuela de Ciencias Biológicas (LEUNA), Universidad Nacional, Heredia, Costa Rica.

#### Distribution.

Colombia, Honduras, Peru (Waltz and McCafferty 1987; McCafferty and Lugo-Ortiz 1996; Massariol et al. 2013; Forero-Céspedes et al. 2016; Kluge 2017), and Costa Rica.

**Figure 8.**
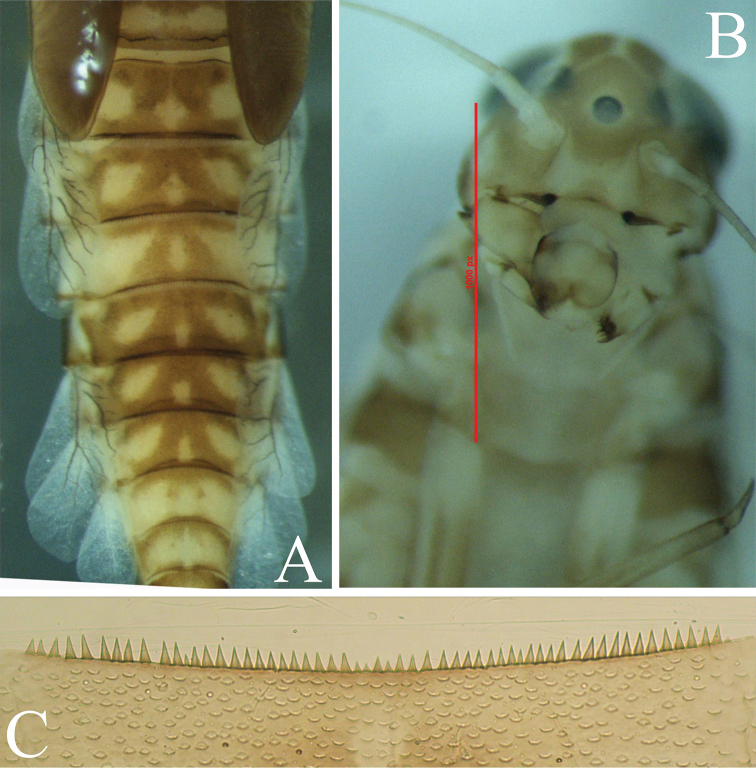
*Cloeodes excogitatus*: **A** abdomen coloration **B** intra-anternal extension (scale:1000 pixels : 2 mm) **C** spines on tergum III.

**Figure 9.**
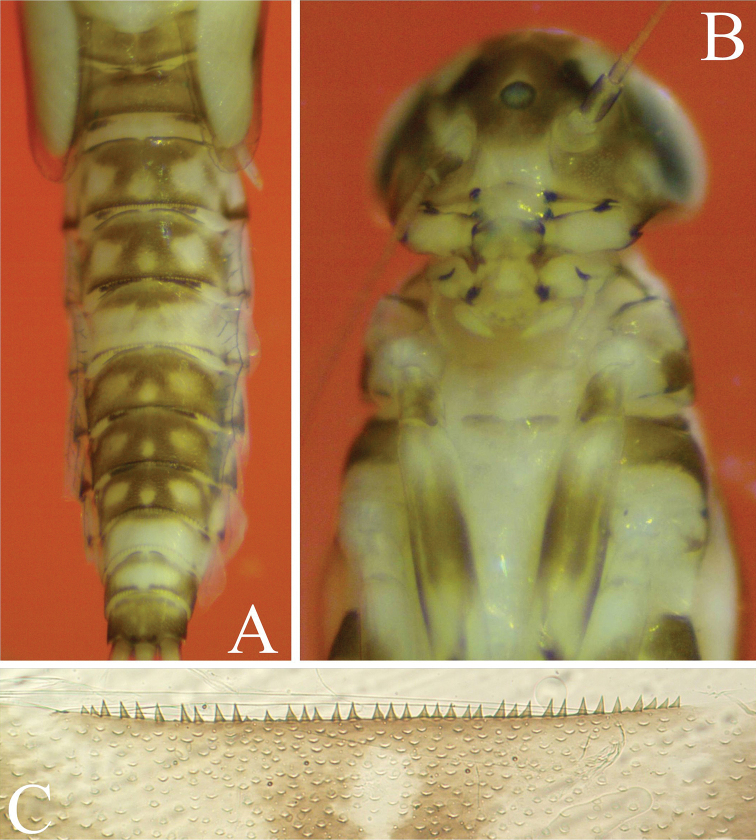
*Cloeodes redactus*: **A** abdomen coloration **B** intra-anternal extension **C** spines on tergum III.

## Discussion

*Cloeodes danta* sp. nov. shares morphological affinities with the following species: *C. caraibensis*Hofmann et al. 1999, *C. excogitatus*, *C. redactus*, and *C. maculipes* Traver, 1938, including, absence of hind wing pads, segment III of labial palp rounded, maxillary palp shorter than galea-lacinia, and the general shape of other structures like labrum, labium and the apex of the femur that were recognized by Hofmann et al. (1999) and Nieto and Richard (2008). Based on the material and literature reviewed, we consider that Central American and Caribbean species can be separated in two groups according to the depth of the central cleft on the right mandible incisives: in *C. excogitatus* and *C. redactus* the cleft breaks down below the level of mandible first inner incisor apex (Waltz and McCafferty 1987: fig. 27; Kluge 2017: fig. 66), while in *C. danta* sp. nov., *C. caraibensis* and *C. maculipes* the cleft breaks down at the level of the first inner incisor apex (Waltz and McCafferty 1987: figs 3, 4 ; Hofmann et al. 1999: fig. 37; Fig. 4C).

In regard to *C. caraibensis* from Lesser Antilles, these species mentioned above have setae on segment III of the labial palp as long as the setae on the glossa and paraglossa (Waltz and McCafferty 1987: fig. 6; Hofmann et al. 1999: fig. 41), while in *C. danta* these setae are shorter (Fig. 5A). Also, labrum intermediate setae are well developed in *C. maculipes* (Waltz and McCafferty 1987: fig. 2), while in *C. danta* sp. nov. they are minute (Fig. 3C); in regard to *C. caraibensis* this character is absent, and this species also shares some diagnostic characters with *C. danta* sp. nov. like scale-bases present on most of the body, shape of III segment of labial palp and spines on the corner of the posterior margins of the sterna (Hofmann et al. 1999: figs 36, 41, 50); however, *C. danta* sp. nov. can be differentiated by no spots or color pattern on the abdomen, right mandible prostheca with two well-developed branches, glossa apically not lobulated, distal portion of gills rounded, minute spines on the terga and sterna margins, and the posterior margin of the paraproct with no bifurcated spines (Figs 2A, 2B, 4C, 4D, 6A–6G, 6K).

Furthermore, *C. danta* sp. nov. shares similar features with *C. excogitatus*, such as the number of spines in the terga III, the shape segment III of labial and the abundance of setae on it (20–25), the number of spines in the paraproct, and body size (Waltz and McCafferty 1987, but *C. danta* sp. nov. can be identified by the different abdominal color pattern, abundant scale-bases throughout most parts of the body, the maxillar palp is equal in length to the galea-lacinia and the absence of a narrow intra-antennal extension. Also, the new species resembles *C. redactus* in the length of the maxillary palp being about as long as the galea-lacinia, the number of spines in the paraproct and the lack of any projections on the apex of the femur (Waltz and McCafferty 1987; Kluge 2017); however it could be distinguished by the shape and number of spines in terga III, the absence of the colorless spots in some terga, a larger body size, the presence of an intermediate setae on the labrum, and the presence of thick spine-like teeth in the inferior corner of sterna III.

In order to improve the identification of *Cloeodes* species in the Central American region, we provide a key to distinguish *C. danta* sp. nov., *C. excogitatus* and *C. redactus*. This will be a useful tool for future aquatic research in the region, which has been increasing over the last 20 years due to development of water quality monitoring using aquatic insects (Alonso-Eguía et al. 2014; Carrie et al. 2017; Castillo 2018; Dávila-Recinos et al. 2019; Pérez 2019).

### Key to mature nymphs of *Cloeodes* species in Central America

**Table:** 

**1**	Abdomen with brownish uniform color pattern, without spots or distinctive marks; body covered with scale-bases; intermediate setae minute; cleft on right mandible incisive breaks down at level of first inner incisor apex and thick spines like teeth present in sternal III corners (Figs 2A, 2B, 4C, 6I, 6J); highlands (2054 m asl)	***C. danta* sp. nov**
–	Abdomen with marks or spotted color pattern (Figs 8A, 9A); body not covered with scale-bases cleft on right mandible incisives breaks down below level of mandible first inner incisor apex; intermediate setae absent; no spines on sternal corners of sterna III (Waltz and McCafferty 1987; Kluge 2017); mid or lowlands	**2**
**2**	Abdominal coloration on terga I–III and V–VII with 3 pale spots (the middle spot being smaller); intra-antennal extension narrow, labrum arc of anterodorsal setae with 5 simple setae; segment III of labial palp ventrally with 20–25 setae; maxillary palp shorter than galea-lacinia; 30–35 spines on tergum III (Fig. 8B, C; Waltz and McCafferty, 1987: figs 25, 36, 40); midlands (1063 m asl)	***C. excogitatus***
–	Abdominal coloration with terga I–III and V–VII with dark brown transverse stripes on posterior margins; intra-antennal extension not narrowed; labrum arc of anterodorsal setae with 3 simple setae; segment III of the labial palp with 30–35 setae; maxillary palp about same length as galea-lacinia; 15–20 spines on tergum III (Fig. 9B, C; Kluge, 2017: figs 6, 12, 14, 58, 59); lowlands (79 m asl)	***C. redactus***

## Supplementary Material

XML Treatment for Cloeodes danta

XML Treatment for Cloeodes excogitatus

XML Treatment for Cloeodes redactus
